# *In Vivo* Pattern Classification of Ingestive Behavior in Ruminants Using FBG Sensors and Machine Learning

**DOI:** 10.3390/s151128456

**Published:** 2015-11-11

**Authors:** Vinicius Pegorini, Leandro Zen Karam, Christiano Santos Rocha Pitta, Rafael Cardoso, Jean Carlos Cardozo da Silva, Hypolito José Kalinowski, Richardson Ribeiro, Fábio Luiz Bertotti, Tangriani Simioni Assmann

**Affiliations:** 1 Federal University of Technology-Paraná, Pato Branco-PR 85503-390, Brazil; E-Mails: leandro.karam@pucpr.br (L.Z.K.); rcardoso@utfpr.edu.br (R.C.); jeanccs@utfpr.edu.br (J.C.C.S.); hjkalin@utfpr.edu.br (H.J.K.); richardsonr@utfpr.edu.br (R.R.); bertotti@utfpr.edu.br (F.L.B.); tangriani@utfpr.edu.br (T.S.A.); 2 Pontifical Catholic University of Paraná, Curitiba 80215-901, Brazil; 3 Federal Institute-Paraná, Palmas-PR 85555-000, Brazil; E-Mail: christiano.pitta@ifpr.edu.br

**Keywords:** pattern classification, machine learning, ingestive behavior, biomechanical forces, fiber Bragg grating sensor (FBG)

## Abstract

Pattern classification of ingestive behavior in grazing animals has extreme importance in studies related to animal nutrition, growth and health. In this paper, a system to classify chewing patterns of ruminants in *in vivo* experiments is developed. The proposal is based on data collected by optical fiber Bragg grating sensors (FBG) that are processed by machine learning techniques. The FBG sensors measure the biomechanical strain during jaw movements, and a decision tree is responsible for the classification of the associated chewing pattern. In this study, patterns associated with food intake of dietary supplement, hay and ryegrass were considered. Additionally, two other important events for ingestive behavior were monitored: rumination and idleness. Experimental results show that the proposed approach for pattern classification is capable of differentiating the five patterns involved in the chewing process with an overall accuracy of 94%.

## 1. Introduction and Contextualization

The understanding of the processes associated with the forage grazing system is related to the assessment of the food intake and ingestive behavior of animals. This kind of study aims to classify food intake and ingestive processes to support the selection of forage that results in increased weight gain and to assist the processes of growth, production and reproduction [1,2]. It also helps in determining the productivity of pastures, is an important measure of animal impact on pastoral ecosystems and may influence agriculture and precision livestock farming [3]. In addition, the monitoring of food consumption activities provides information on the health and well-being of the animal [2].

An important activity related to animal health and food utilization is the rumination process [4]. This process is related to the gastrointestinal capacity to transform compounds of plant cells, such as cellulose and hemicellulose, into energy. These characteristics of the ruminant digestive system allow the better usage of energy from fibrous plants. The rumination process is strongly related to the type of forage used during the feeding process [4]. This shows that, besides the determination of the type of ingested food, the identification of the rumination process is also important for studies aimed at improving animal handling in order to increase productivity [4,5].

Different techniques have been used to assess the ingestive behavior of animals in grazing environments. Among these techniques, some are based on the evaluation of the animals digestive behavior [6], while other techniques use mechanical sensors [7,8] or are based on acoustic methods [9,10]. Since it is not invasive and has a low cost, the acoustic method is the primary technique employed [2,9]. This technique uses audio sensors to obtain data on mandibular movements of animals during the grazing period. However, the audio signal can be affected by interference from external sources that are not related to the chewing process, and like all of the others, the analysis and classification of the data obtained are performed manually [9,10]. Computational tools for this purpose are scarce and have a success rate for classification of approximately 84%, including at most four classes [11].

The acquisition and analysis of chewing movements in ruminants allow the characterization of chewing patterns with high immunity to noise and the development of a computational system that provides greater automation and a higher success rate in the stage of classification, which are important for further advances in equipment for the evaluation of animals’ feeding behavior. Thus, this paper proposes a new paradigm for the monitoring and classification of chewing patterns. In order to provide interference immunity to the signal acquisition stage, fiber Bragg grating sensors (FBG) are employed as the transducer element [12]. For the automation of the classification process, a classifier based on decision trees is proposed and developed [13].

The optical biocompatible sensors [14] used in this work were developed from a new technique of packaging with preliminary *in vitro* tests [15], where the viability of the technique has been demonstrated. Furthermore, with the objective of developing a package for the optical sensor, tests were performed *ex vivo* [16]. The work resulted in a totally biocompatible sensor without body rejection or tissue reaction due to the fact that its material (silica) is not toxic. Besides that, the sensor is chemically stable, immune to electromagnetic interference, has reduced dimensions with a diameter and a length in the order of micrometers and millimeters, respectively, and provides excellent sensitivity for the acquisition of low intensity signals. Another feature is that it is suitable for monitoring geometrically-irregular regions, such as cheekbones, where it is difficult to apply conventional electric extensometry [17]. These characteristics make FBGs suitable for *in vivo* experiments [18,19].

The improvement in the data classification process is obtained with the use of machine learning techniques. One possibility is the use of inductive learning techniques, such as decision trees, neural networks, support vector machines, genetic algorithms, and so forth [20]. One of these techniques, the decision tree algorithm C4.5, is known for its capacity of generalization of data susceptible to noise and for working with continuous and categorical attributes, and it also handles missing attribute values [13]. In addition, classifiers constructed with decision trees have a good combination of the speed and error rate of classification [21]. These characteristics make the decision trees a natural choice for the problem in question and have been employed successfully in different areas requiring the classification of patterns, such as in the areas of medical [22,23], safety [24], chemistry [25], geology [26], among other areas. Despite the success of such approaches, there are few studies related to the use of machine learning techniques applied in classification problems of ingestive behavior patterns of ruminants.

Studies for the assessment of ruminant feeding behavior using FBG arose from *in vitro* [15] and *ex vivo* [16] experiments. Therefore, these sensors allow the evaluation of strain applied by the animal along time for each chewing movement. In order to improve the classification process of the acquired data, the signals were segmented into each chewing movement, and the frequency spectrum of the signals of each chewing movement was also analyzed to include new features in the dataset.

The description of the proposed instrumentation and classification of chewing patterns of ruminants is organized as follows: Section 2 describes the proposed instrumentation and algorithms used to classify the acquired data. Section 3 presents the pre-processing of the acquired data and the creation of the training set. In Section 4, the results are presented and discussed. Finally, Section 5 concludes the paper. This work has the approval of the Ethics Commission on the use of Animals of the Federal University of Technology-Paraná (CEUA 2013-009 Protocol).

## 2. Materials and Methods

### 2.1. Instrumentation and Data Acquisition

In this work, the mandibular stress is measured by an FBG sensor with $\lambda_{B}$ = 1541 nm; reflectivity = 50%; FWHM (full width at half maximum) = 0.3 nm. The sensor was set on a surgical titanium plate with cyanoacrylate glue. The plate was attached to the animal’s jaw by surgical screws. The animal used was a calf of 2 months of age, weighing approximately 160 kg. All of the materials used in the surgical process are biocompatible, and there is no rejection by the animal after the deployment of the sensor. A detailed description about the employed sensor can be found in [16].

The access to the mandible was made through a surgical procedure, through an incision of the skin and the masseter muscle, in order to reach the masseteric tuberosity, over which the sensor is placed. The location of the instrumentation was based on and described in [15,16], which defined the position of the sensor to better capture the strain generated from masticatory processes of different materials.

**Figure 1:**
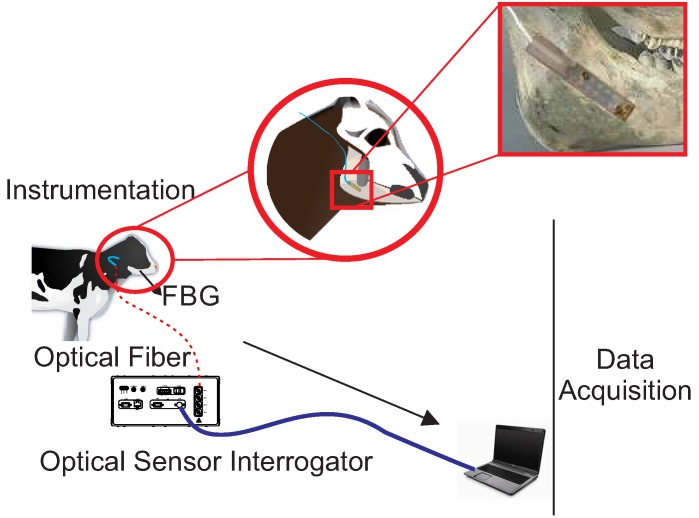
Positioning of the sensor and data acquisition.

For data acquisition, the interrogator DI410 was used, manufactured by HBM^®^, along with CatmanEasy^®^ software. The sampling rate used in the tests was 1 kS/s. Figure 1 illustrates the positioning of the sensor and the *in vivo* data acquisition process proposed in this work.

For the acquisition of data during chewing, different types of food were provided to the animal. The first food provided was dietary supplement in the form of pelletized concentrate with an intake time of 13 min. The second material provided to the animal was Tyfton (Cynodon) hay, with an ingestion time of approximately 10 min. Additionally, the third food provided was ryegrass (Lolium multiflorum), which was consumed for about 5 min. During the period of food intake, there were intervals without mastigatory movements. These intervals were used for collecting the idleness class samples. Samples of this class were also obtained between the end time of feeding and the beginning of rumination. After approximately 45 min from the end of food collection, the process of rumination began, which was monitored for about 15 min. The acquired data were used as a training set for the machine learning algorithm described in the next section.

### 2.2. Decision Tree C4.5 Algorithm

The choice of decision trees over the artificial neural networks was made due to the insertion of decision tree algorithms into the symbolic learning paradigm, which allows a clearer view of the created set of rules. It also makes clear the key attributes used in the decision tree structure. The decision tree algorithms also perform well when working with a large number of forecaster attributes.

A decision tree has a structure composed of nodes, branches from these nodes, and terminal nodes (leaf nodes). The nodes represent the attributes of an instance. The branches receive the possible values of the attribute in question, and the leaf nodes represent the different classes present in the dataset. The classification consists of following the path determined by successive nodes arranged along the tree until a leaf node is reached, which contains the class to be assigned to the respective instance [13]. In this work, the C4.5 algorithm was used [13], which provides enhancements to the ID3 algorithm [27], where the ability to work with categorical and quantitative attributes was added.

For the construction of the decision tree, a dataset *D* is taken into consideration with *m* instances, $D=\left\{{\bar{D}}_{1},{\bar{D}}_{2},\cdots ,{\bar{D}}_{m}\right\}$, where each instance ${\bar{D}}_{i}$, i=1,…,m, is a subset with *n* attributes $a_{ij}$, where j=1,2,…,n, *i.e.*, ${\bar{D}}_{i}=\left\{a_{i1},a_{i2},\cdots ,a_{in}\right\}$. The set of possible values for attribute $a_{ij}$ is represented by $dom\left(a_{ij}\right)=\left\{v_{1},v_{2},\cdots ,v_{k}\right\}$, where $v_{l}$, l=1,…,k, are the possible values for attribute $a_{ij}$. Each instance of the dataset *D* is classified according to a set of classes $C=\left\{c_{1},c_{2},\cdots ,c_{w}\right\}$, where *w* is the number of classes.

Algorithm C4.5 uses the attributes that generate a higher information gain ratio to create the node of the decision tree. The evaluation of the information gain ratio depends on the information gain [27]. The information gain uses as a basis a measure known as entropy [13,27]. The entropy measures the amount of information needed to identify the class of a node [28].

Based on the computation of information gain and the information gain ratio of each attribute, the attribute with the highest information gain ratio is selected as the node attribute [13]. For each possible value of the node, branches are created. If all instances belong to the same class, the branch points to a leaf node, which characterizes the classification. Otherwise, the calculations are recursively performed until all instances can be classified.

## 3. Training Set Creation

Prior to the creation of the training set, some pre-processing of the acquired data is necessary to extract as much information as possible from the signal. After the pre-processing, the training set can be created. This section covers these topics.

### 3.1. Data Pre-Processing

The acquired data, corresponding to the classes of interest, are labeled according to the type of food consumed by the animal or according to the ingestive event during the signal acquisition. The foods provided to the animal along with the idleness and rumination events form five classes that compose the set of target attributes *C*, that is,

(1) C={dietarysupplement,hay,ryegrass,rumination,idleness}

**Figure 2:**
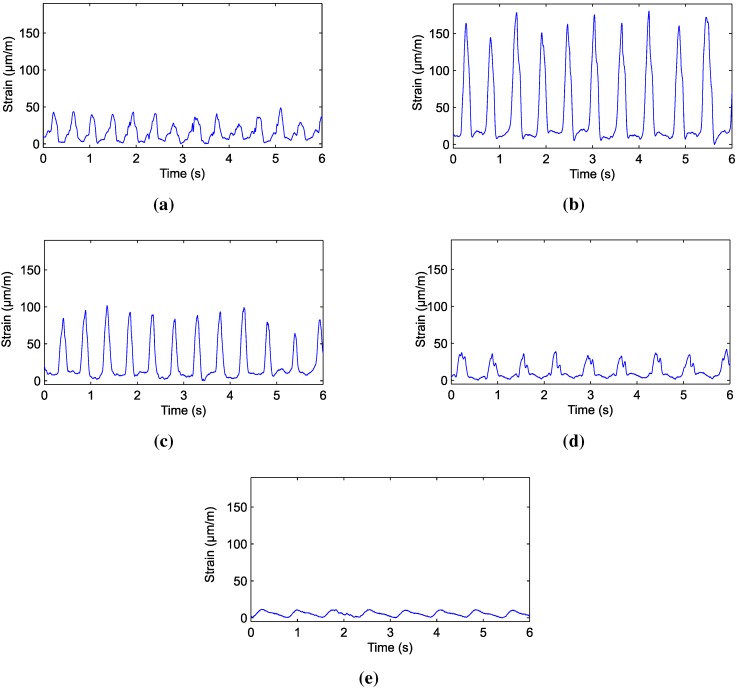
Chewing signals acquired: (**a**) dietary supplement; (**b**) hay; (**c**) ryegrass; (**d**) rumination and (**e**) idleness.

Figure 2 presents 6 s of an already labeled set of data. It is observed that each of the items shows a specific waveform, as well as distinct strain values. This is due to the texture of the forage used, the dietary supplement and the bolus present in rumination. The peaks in Figure 2 express the maximum aperture of the mouth, while the minimum values represent the closure of the animal’s jaw.

In order to classify the chewing movements, the signal should be segmented to obtain a particular sample for each movement. To segment the signal, firstly, the average value of the signal is evaluated every second. Then, the average is subtracted from the original signal and zero-crossing detection is used to detect the beginning and the end of each movement. After the segmentation, the signal that describes the jaw movement is again taken into account with the average value added.

It was defined that the data collected of each jaw movement during 1 s form an instance of the training set. For the classification system, each sample of the signal is an attribute. Considering the sampling rate of 1 kS/s, all of the training set has 1000 attributes by chewing movement. Since, after the segmentation, the instance of the training set can have less then 1000 attributes, if this is the case, the average value of the signal is reinserted in the set to provide 1000 elements. The choice of the average value of the signal to the complementation of the attributes is justified, since it is information that varies for different classes, which contributes to a more effective classification.

**Figure 3:**
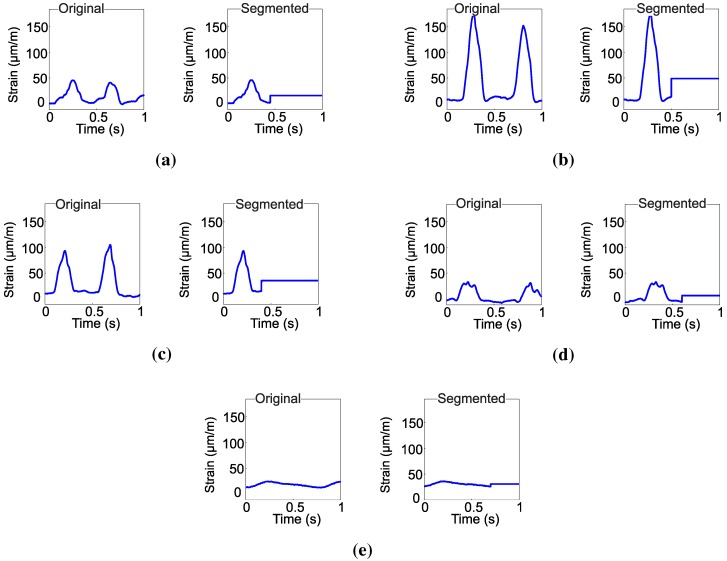
Original and segmented chewing signals for: (**a**) dietary supplement; (**b**) hay; (**c**) ryegrass; (**d**) rumination and (**e**) idleness.

Finally, each chewing movement, considering its average, is labeled according to the class associated with the respective event. Figure 3 illustrates the segmented waveforms of each movement considering its average value.

**Figure 4:**
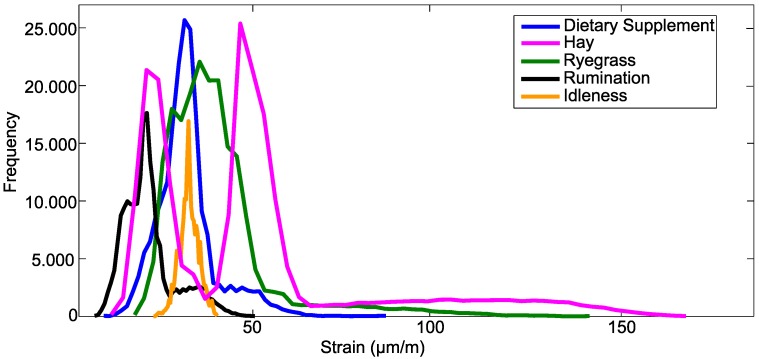
Histogram of the dataset.

Figure 4 shows the histogram of the strain values of the entire training set. It is observed that the distribution of values of strain between classes is quite distinct. For example, rumination and idleness have strain values concentrated in a small range of values. For rumination, the strain value was 42 µm/m. The maximum strain in the bone tissue for the idleness class was in the order of 12 µm/m. The waveform of these materials is also quite distinct. These behaviors noticed in the signal of each material can improve the classification procedure.

However, other events have strain values ranging in a larger interval of values, such as hay, dietary supplement and ryegrass. Chewing movements performed with hay induced larger wavelength shifts, which resulted in a strain of 178 µm/m. This behavior is different from the other classes, which also helps the separation of the classes during the classification stage. However, most of the movements performed with dietary supplement and ryegrass have maximum strain in the order of 50 µm/m and 100 µm/m, respectively. When there are events with values of a similar wavelength, difficulties can occur in the separation of the classes.

Therefore, in order to extract additional information of the signals, the use of the fast Fourier transform (FFT) is proposed for the extraction of relevant information from the frequency spectrum of the signal [29]. Therefore, there is indirect information about the shape of the acquired signal that is suitable to use with decision trees. The frequency spectrum is obtained through [30]:

(2) $$X\left(k\right)=\frac{1}{N}\sum_{n=0}^{N-1}x\left(n\right)e^{-j\frac{2\pi }{N}kn},\;\;\;0\leq k\leq N-1$$

where *N* is the number of samples contained in the signal under analysis. In this case, it corresponds to the number of samples in one second, that is N=1000.

### 3.2. Creation of the Training Set

For creating the training set, 200 instances were used for each class, totaling 1000 instances. Each instance ${\bar{D}}_{i}$ has 1030 attributes, where the first 1000 attributes come from the segmentation of the data of a chewing movement that was described in Section 3.1. The 30 additional attributes are formed by the first 30 components of the frequency spectrum of the signal obtained by calculating the FFT. In this way, the training set *D* is given by:

(3) $$D=\left\{{\bar{D}}_{1},{\bar{D}}_{2},\cdots ,{\bar{D}}_{m}\right\}$$

where m=1000. Each element ${\bar{D}}_{i}\in D$ has an associated label. This label defines the class to which the instance belongs:

(4) $${\bar{D}}_{i}=\left(\vec{x_{j}},y_{z}\right)$$

in which $\vec{x_{j}}$ is a vector of 1030 elements with the values representing the attributes of the instance ${\bar{D}}_{i}$, $y_{z}$ is the value of the class associated with this instance and $y_{z}\in C$.

**Figure 5:**
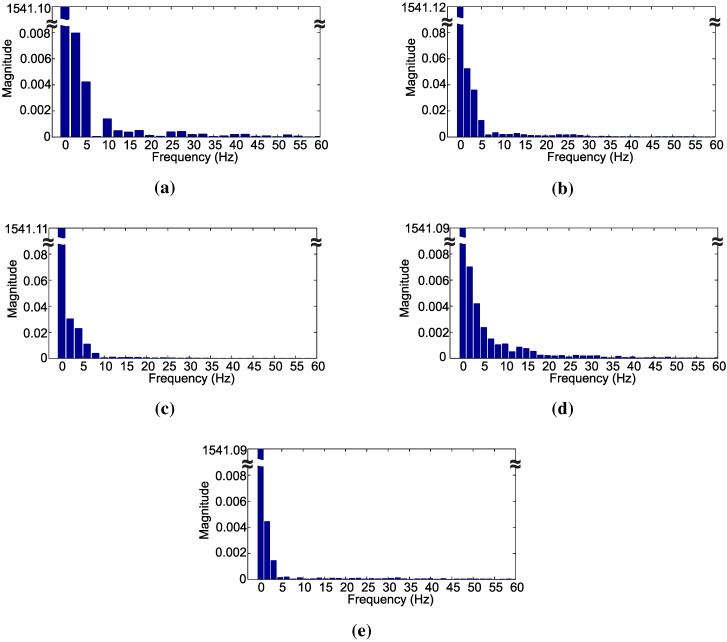
Frequency spectrum of chewing signals for: (**a**) dietary supplement; (**b**) hay; (**c**) ryegrass; (**d**) rumination and (**e**) idleness.

Figure 5 shows the frequency spectrum of the signals representing a chewing movement of each class. The frequency spectrum shows that different frequency components can be noticed for each pattern. Frequency components above the 29th harmonic almost vanish, and its use in the decision algorithm did not produce any further performance improvement.

In Figure 5a–e, the frequency bin spacings are 2.49 Hz, 1.66 Hz, 2 Hz, 1.66 Hz and 1.54 Hz, respectively. Different bin amplitudes reflect in distinct waveforms, while different bin spacing relates to different periods of the signal. Hence, the use of the frequency spectrum can be valuable in the identification of various signal waveforms related to distinct chewing movements.

Comparing the frequency spectrum of the signals presented in Figure 5 with the signals in the time-domain depicted in Figure 2, it is evident that the frequency bin spacing represents the period of the analyzed signals.

The first bin, that is the DC component of the signal, is used as the 1001st attribute, while for the second, up to the 29th harmonic components are also used as attributes. The frequency of each harmonic component depends on the chewing pattern, as mentioned above. Therefore, 30 extra attributes are extracted from the signal that are used in conjunction with the 1000 attributes that came from the original signal to form the 1030 attributes used in the training.

After the creation of the training set, it was submitted to the decision tree algorithm C4.5, described in Section 2.2, for the generation of the decision tree.

## 4. Results and Discussion

During the generation of the decision tree, k-fold cross-validation was used [31]. k-fold cross-validation evaluates the ability of the generalization of the set of rules generated for the decision tree.

In k-fold cross-validation, the dataset is divided randomly into *k* subsets. Of these, k-1 subsets are used for training, and one subset is used for testing. This process is repeated *k* times, with each of the *k* subsets used exactly once as the test set. In this way, different classifiers are obtained, and the accuracy of the training and testing sets can be evaluated. After the tests, the classifier that provided the best accuracy was selected. The tests were carried out using 10-fold cross-validation.

To reduce the risk of overfitting of the decision tree, the algorithm uses the post-pruning method [13,32]. The post-pruning aims to generalize the decision tree that was generated in the training phase by generating a subtree that avoids overfitting of the training data. The post-pruning consists of removing nodes of the tree that do not contribute to its generalization ability. Pruning reduces the complexity of the induced set of rules, improving the accuracy and reducing the overfitting [33,34].

**Figure 6:**
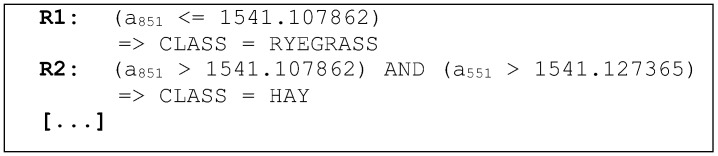
Example of the set of rules generated by the classifier.

The knowledge obtained in the application of the machine learning technique is represented by a set of ordered rules, which are shown in Figure 6. Rule R1 is read as follows: “IF (the $a_{851}$ attribute value is less than or equal to the 1541.107862 nm) then (ryegrass class)”.

**Figure 7:**
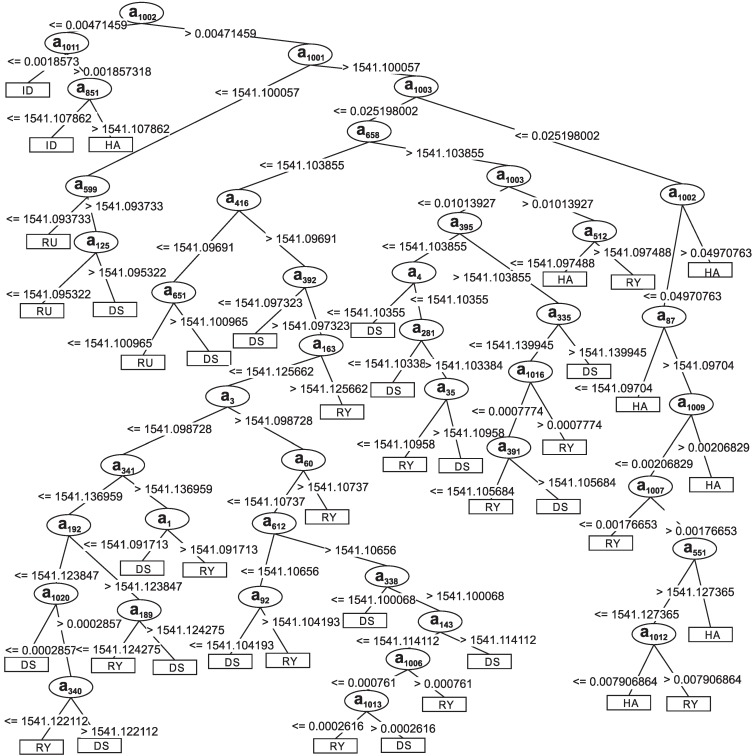
Decision tree generated using the training set *D*. DS = *Dietary Supplement*; HA = *Hay*; RY = *Ryegrass*; RU = *Rumination*; ID = *Idleness*.

Figure 7 represents the decision tree generated from the training set *D*, presented in Section 3.2. The classifier is composed of 83 nodes, 42 leaf nodes and 41 decision nodes. It should be highlighted that the first to the 1000th attributes are related to the acquired signals, while the 1001st to 1016th attributes come from the frequency spectrum obtained through the FFT calculation. The 1002nd divided the tree into two subtrees. The first one classified all instances of idleness. This attribute refers to information obtained with the introduction of the frequency spectrum. The second subtree has decision nodes composed of original signal-related attributes as informational attributes from FFT. Among the subdivisions of the decision tree, the subtree formed by attribute 1001 can also be highlighted, which contains the average value of the signal. This node sorted most of the instances of the class dietary supplement.

The frequency components of the signals were used to compose the structure of the decision tree presented in Figure 7. Observing the decision tree, the first node of the tree is the fundamental frequency component $a_{1002}$. This node is responsible for separating the idleness class from the other classes. This class has the lowest fundamental frequency, that is 1.54 Hz. Hence, all of the idleness events are classified near the starting node.

Another important node is related to the attribute $a_{1001}$. This node corresponds to the DC level of the signals and separates the rumination class from the others. The rumination class has the lowest DC value among the classes dietary supplement, hay and ryegrass, as can be seen in Figure 2. In fact, the DC level of the idleness class is lower than rumination, but the fundamental frequency, node $a_{1002}$, was already responsible for its classification.

The second harmonic component, node $a_{1003}$, separates the tree into two subtrees. One subtree is responsible for classifying the classes hay and ryegrass. Inside this subtree, the decision nodes is associated with the information that came from the frequency spectrum. The second subtree that starts at node $a_{1003}$ classifies the classes dietary supplement and ryegrass. In the subtrees that are derived from this point, a great part of the decision nodes is associated with the attributes that came from the sampled signal.

The data presented in Figure 2, Figure 3 and Figure 4 show the strain of the bone tissue during chewing movements. However, the dataset used during the training of the decision tree classifier was formed by wavelength values collected by the optical interrogator, since one of the objectives of the study was the classification of chewing movements in real time. Then, the rules shown in Figure 7 were formed by wavelength values.

Table 1 displays the confusion matrix with the classification results of the training set *D*. The generated classifier provided a higher accuracy for rumination and idleness classes. These are classes that have values of wavelength and waveform also distinguished from the others. The classes dietary supplement and ryegrass provided the lowest accuracy. Changes in strain values of these foods are similar to the values in some samples of chewing. For dietary supplement, the strain is approximately 50 µm/m, while ryegrass had instances with strain in the order of 60 µm/m. It is also noticed that some samples of these classes have a similar waveform, which results in incorrect classification of some instances.

**Table 1:** Confusion matrix for results obtained through the algorithm C4.5 using the training set *D*. (1) dietary supplement; (2) hay; (3) ryegrass; (4) rumination; (5) idleness.

	1	2	3	4	5
1	172	0	24	4	0
	(86%)	(0%)	(12%)	(2%)	(0%)
2	4	196	0	0	0
	(2%)	(98%)	(0%)	(0%)	(0%)
3	24	4	172	0	0
	(12%)	(2%)	(86%)	(0%)	(0%)
4	0	0	0	200	0
	(0%)	(0%)	(0%)	(100%)	(0%)
5	0	0	0	0	200
	(0%)	(0%)	(0%)	(0%)	(100%)

The average success rate was 94%, *i.e.*, of the 1000 training set instances, 940 were correctly classified during the process of the generating and testing of the decision tree. This success rate supplants success rates of other methods available in the literature, which are around 84%, where only four classes were considered [11].

Other similar tests were also conducted with smaller numbers of forecaster attributes. In a second test, 500 attributes of the original signal were used along with the same frequency components that were previously described. In this test, the accuracy was 92%. Another test using 250 attributes of the original signal with the same frequency components resulted in an accuracy of 89.5%. It can be seen that even with a reduced number of forecasters attributes the accuracies are around 90%, which can be considered satisfactory when compared to other techniques in the literature that provide 84% accuracy.

Previous works had already discussed the use of optical sensors in other biomechanical applications where the bone strain must be measured [16,35,36]. However, no *in vivo* studies were executed. The experiments presented in this study were conducted in one animal due to the restricted availability of animals for study. Nevertheless, the technique can be applied in other animals, providing new data for processing. With additional data, it is possible to include different information that can be used during the training and validation tests of the classification system aiming at a decision tree with better generalization characteristics.

## 5. Conclusions

This work presented the development of a system for the classification of chewing patterns in ruminants. This system can bring significant improvements to the studies related to animal nutrition and precision livestock farming.

The proposed approach was based on machine learning using the decision tree algorithm C4.5. The data provided to the classifier were obtained through *in vivo* optical extensometry using fiber Bragg grating sensors. The sensor used has high sensitivity, and at the same time, it is immune to electromagnetic interference and offers excellent features of biocompatibility.

It was shown that the application of FBG sensors to collect ruminant nutrition-related data is promising. The sensor response is capable of showing the dependence of the chewing force (through the bone strain) and frequency on the hardness and fibrous contents of the given food.

The obtained classifier provided a high success rate due to the pre-processed data. The pre-processing includes information on the frequency spectrum of the signals.

The achieved results showed that the decision tree technique can be used to generate a classifier trained with data from different types of chewing patterns. The classifier was also able to identify other events related to animal nutrition, such as idleness and rumination. The latter one is an important source of data for animal health and welfare monitoring.

The induced set of decision rules was capable of generalizing most patterns in an appropriate manner. The overall success rate average was 94%. Success rates significantly exceed those observed in the literature, where the classifiers work with a maximum of four different classes, and the percentage of success is approximately 84%. Moreover, the technique presented in this paper, after the proper training, allows for greater automation of classification when compared to the usual methods.
